# Vagal nerve stimulation in myocardial ischemia/reperfusion injury: from bench to bedside

**DOI:** 10.1186/s42234-024-00153-6

**Published:** 2024-09-13

**Authors:** Giuseppe Giannino, Lorenzo Nocera, Maria Andolfatto, Valentina Braia, Federico Giacobbe, Francesco Bruno, Andrea Saglietto, Filippo Angelini, Ovidio De Filippo, Fabrizio D’Ascenzo, Gaetano Maria De Ferrari, Veronica Dusi

**Affiliations:** 1 Cardiology, Department of Medical Sciences, University of Turin, Torino, Italy; 2 Division of Cardiology, Cardiovascular and Thoracic Department, ‘Città della Salute e della Scienza’ Hospital, Corso Bramante 88, Turin, 10126 Italy

**Keywords:** Ischemia/reperfusion injury, Transcutaneous vagal nerve stimulation, Vagus nerve, Tragus, Bioelectronic medicine, Autonomic imbalance, Cholinergic anti-inflammatory pathway

## Abstract

The identification of acute cardioprotective strategies against myocardial ischemia/reperfusion (I/R) injury that can be applied in the catheterization room is currently an unmet clinical need and several interventions evaluated in the past at the pre-clinical level have failed in translation. Autonomic imbalance, sustained by an abnormal afferent signalling, is a key component of I/R injury. Accordingly, there is a strong rationale for neuromodulation strategies, aimed at reducing sympathetic activity and/or increasing vagal tone, in this setting. In this review we focus on cervical vagal nerve stimulation (cVNS) and on transcutaneous auricular vagus nerve stimulation (taVNS); the latest has the potential to overcome several of the issues of invasive cVNS, including the possibility of being used in an acute setting, while retaining its beneficial effects. First, we discuss the pathophysiology of I/R injury, that is mostly a consequence of the overproduction of reactive oxygen species. Second, we describe the functional anatomy of the parasympathetic branch of the autonomic nervous system and the most relevant principles of bioelectronic medicine applied to electrical vagal modulation, with a particular focus on taVNS. Then, we provide a detailed and comprehensive summary of the most relevant pre-clinical studies of invasive and non-invasive VNS that support its strong cardioprotective effect whenever there is an acute or chronic cardiac injury and specifically in the setting of myocardial I/R injury. The potential benefit in the emerging field of post cardiac arrest syndrome (PCAS) is also mentioned. Indeed, electrical cVNS has a strong anti-adrenergic, anti-inflammatory, antioxidants, anti-apoptotic and pro-angiogenic effect; most of the involved molecular pathways were already directly confirmed to take place at the cardiac level for taVNS. Pre-clinical data clearly show that the sooner VNS is applied, the better the outcome, with the possibility of a marked infarct size reduction and almost complete left ventricular reverse remodelling when VNS is applied immediately before and during reperfusion. Finally, we describe in detail the limited but very promising clinical experience of taVNS in I/R injury available so far.

## Introduction

Despite substantial improvement in surgical and percutaneous revascularization techniques, myocardial infarction (MI), defined as the irreversible damage caused by severe and sustained myocardial ischemia, and the chronic heart failure (HF) which may follow, remain among the leading causes of morbidity and mortality worldwide (Moran et al. 2014). The size of the infarcted area is one of the major determinants of the prognosis of these patients; accordingly, the main goal of cardioprotective strategies is to reduce its extension.

Although restoring coronary perfusion, therapeutically known as reperfusion, is the only strategy able to prevent the progression from ischemia to infarction, it may itself contribute to worsening tissue damage due to the so called ischemia/reperfusion (I/R) injury (Heusch 2015; Hausenloy et al. 2017; Hausenloy and Yellon 2013). As such, therapeutic interventions able to protect the heart against acute I/R injury, to reduce myocardial infarct size and to finally prevent the onset of HF (F, B., et al. 2023; Elia et al. 2024) in patients presenting with acute MI are highly needed. Unfortunately, despite the promising results of several cardioprotective interventions tested in animal models so far, the clinical translation has been challenging (Lecour et al. 2021; Caccioppo et al. 2019) and no such strategy is recommended by current guidelines (Byrne et al. 2023). Specifically, stem cell-based therapies offer huge potential, but their clinical implementation is still limited by engraftment-related side effects (Pezhouman et al. 2023); accordingly, paracrine mediators, including exosomes and microvesicles, are currently under study with cardioprotective purpose (Caccioppo et al. 2019; D’Ascenzo et al. 2021; Femminò et al. 2021).

In the present review we will focus on cervical vagal nerve stimulation (cVNS) and on transcutaneous auricular vagus nerve stimulation (taVNS), being the latest one of the most promising, not cell-based, cardioprotective strategies, currently available; we will span from the pathophysiological rationale to the modalities of use and application in the context of I/R injury. The potential benefit in the setting of post cardiac arrest syndrome (PCAS) will also be mentioned.

## Pathophysiology of ischemia/reperfusion injury

The main pathophysiological feature of I/R injury is the overproduction of reactive oxygen species (ROS) as a result of a number of complex mechanisms including metabolic changes (Tian et al. 2023) and mitochondrial dysfunction (Kuznetsov et al. 2019), cell death (Cell Biology of Ischemia/Reperfusion Injury - PMC n.d.), microvascular obstruction (Sezer, et al. 2018) and inflammation (Zhou et al. 2015; Maxwell and Lip 1997; Thapalia et al. 2014), autonomic imbalance and autonomic remodelling (Intachai et al. 2018; Lambert, et al. 2016). All these mechanisms are deeply related one to the other and are mutually potentiated. Specifically, the tight association between the autonomic nervous system and the immuno-inflammatory responses, better known as neuroimmune crosstalk (Tarnawski and Olofsson 2021), will be discussed in detail due do its relevance for the I/R injury. The neuroimmune crosstalk includes but is not limited to, the cholinergic anti-inflammatory pathway.

### Metabolic changes

Acute myocardial ischemia is the result of an abrupt mismatch between coronary blood flow and metabolic requirements, leading to inadequate oxygen and metabolic substrates delivery. In turn, oxygen and ATP shortage represses oxidative metabolism of fatty acids (FA), carbohydrates, ketones, and amino acids, and activates anaerobic glycolysis. During reperfusion intracellular pH tends to progressively normalize thanks to oxygen wash‐in combined to ischemic metabolites wash-out, leading to the progressive switch back to oxidative metabolism and to ongoing changes in glycolysis. These metabolic changes occurring during ischemia and early reperfusion largely determine the actual infarct size that follows an ischemic episode. Accordingly, several tailored metabolic modulation strategies have been attempted against I/R injury, starting in 1962 with potassium‐insulin‐glucose administration during MI (Sodi-Pallares et al. 1962); most of them failed in translation. For instance, in experimental models, the partial inhibition of myocardial FA oxidation with drugs such as oxfenicine, ranolazine, and trimetazidine stimulates glucose oxidation and reduces lactate production during ischemia (Stanley 2004) but it has not been translated in a significant clinical benefit so far in the acute setting (Morrow 2007).

### Mitochondrial dysfunction

ATP depletion leads to the malfunction of the electrogenic sodium–potassium pumps in the sarcolemma, resulting in an initial cytoplasmic accumulation of sodium. The excess sodium is then extruded through the Na/Calcium exchanger (NCX), resulting in calcium overload, and the Na/H exchanger (NHE1), resulting in acidosis, which is further worsened by the reduced energy efficiency of anaerobic metabolism. These two conditions, beyond dissipating the transmembrane potential and leading to partially depolarized ischemic cells more prone to arrhythmias, induce a profound mitochondrial dysfunction characterized by increased mitochondria permeability through the opening of mitochondrial permeability transition pores (mPTP), increased ROS production and release of cytochrome c, and further reduced ATP generation. Restoring oxygen delivery in the dysfunctional mitochondria with perfusion further worsens ROS production and increases the proteolytic activity of calpain, a calcium activated cytosolic protease whose activation promotes apoptosis. Zinc and Magnesium, as well NCX and NHE1 inhibitors have all demonstrated potential beneficial effects in pre-clinical models of I/R injury through membrane stabilization and indirect mitochondrial protection (Skene et al. 2019; Martens et al. 2022).

### Cell death

The fate of the ischemic myocardium potentially follows three paths: recovery, remodelling, and death. In the context of cell death, we first distinguish necrosis (Mishra, et al. 2019), where cell death results in rupture of mitochondria and cell membrane. Mechanisms contributing to necrosis are failure of ion pumps, acidosis, calcium overload (Tani and Neely 1989), and irreversible damage to proteins, lipids, and nucleic acids as a result of excessive ROS production.

Reperfusion typically intensifies the morphological features of necrosis, with the appearance of contraction bands (Schluter et al. 1996), due to the excessive and uncoordinated contraction produced by abnormal Ca cycling between sarcoplasmatic reticulum and the cytosol, along with an early leukocyte infiltrate. The second type of death is the programmed cell death, which can be described as apoptosis (Davidson et al. 2020), necroptosis (Zhou and Yuan 2014), and pyroptosis (Audia et al. 2018), respectively, depending on the mechanism that causes it. In the first case, the release of cytochrome c from mitochondria promotes activation of caspases with DNA fragmentation; in the second, the activation of tumor necrosis factor receptors (TNFRs) or toll-like receptors (TLRs) promotes the formation of sarcolemma pores; in the third, the release of damage-associated molecular pattern molecules (DAMPs) promotes the formation of inflammasome and gasdermin-dependent pores in the sarcolemma. In the latter two cases, as in necrosis, the loss of cell membrane integrity promotes the inflammatory response.

The mechanisms of protection from cell death can in turn be categorized into three main pathways, PKC-eNOS-PKG; RISK and SAFE pathways, the activation of which may potentially be beneficial in models of I/R injury (Amoretti et al. 2002).

### Inflammation and microvascular obstruction

Activation of the innate and adaptive autoimmune system plays a major role in both I/R injury and the healing pathway. The innate response is triggered by the release of DAMPs from necrotic myocardium or gone through cardiomyocyte membrane portioning, through predominantly TLR2 and TLR4, which are broadly expressed in cardiac tissue (Vilahur and Badimon 2014).

After activation, the signalling begins via TRIF (TIR-domain-containing adaptor-inducing beta interferon) and MyD88-dependent pathways resulting in IFN3 and NFκB activation, respectively, and subsequent production of pro-inflammatory cytokines (e.g. IL-1, IL-6, and Tumor necrosis factor- alpha, TNF-α) (Meijerink et al. 2015), promoting the infiltration of neutrophils into the site of cardiac injury to initiate inflammatory responses.

In addition, the release of DAMPs promotes platelet activation, endothelial damage (Silvis et al. 2020) and the occurrence of microvascular thromboembolic events, which, together with endothelial dysfunction, promote microcirculation no-reflow after reperfusion. Microthrombi also, in turn, play a critical role in leukocyte migration to the ischemic region, amplifying the inflammatory burden (Ghasemzadeh et al. 2022).

The complement system is also involved in the innate inflammatory response; in fact, C1 inhibitors and inhibitors of mannose-binding protein-associated serine protease 2 (MASP2), two regulators of complement activation, have been shown to be effective in preclinical studies.

The adaptive immune system, including T and B lymphocytes, play a central role in infarct size extension (Flohr and Breull 1975). CD4+ T lymphocytes increase myocardial I/R injury by the production of pro-inflammatory cytokines like interferon-γ (IFNγ) and TNFα (Hofmann and Frantz 2015). Otherwise, CD4+ CD25+ regulatory T cells (Tregs), a specific subset of CD4+T lymphocytes, can beneficially control myocardial I/R injury. Treg cells exert their immunomodulatory function through two different mechanisms, including cell- contact-dependent mechanisms and the production of anti-inflammatory cytokines like IL-10 and transforming growth factor (TGF)-β, involved, together with endothelial integrity preservation, in remodelling of heart muscle and atherosclerotic plaque (Candreva 2024).

### Autonomic imbalance and autonomic remodelling

I/R injury is associated with a profound autonomic imbalance characterized by sympathetic overactivity and parasympathetic withdrawal, that plays a pivotal role in the progression from ischemia to infarction, as well as in the associated risk of arrhythmias and of evolution towards heart failure (Gronda, et al. 2022).

The primum movens for this imbalance is characterized by an abnormal neuronal afferent signaling sustained by chemo and mechanoreceptor stimulation within the ischemic area due to, respectively, ischemic metabolites (mostly adenosine, but also lactic acid, bradykinin, prostaglandins, and ROS) and abnormal contraction leading to increased wall stress (Dusi and Ardell 2020). Mechano-transduction, that is mostly mediated by myelinated (A delta type) fibers, occurs fast (within few seconds after acute coronary occlusion), produces phasic activity in the afferent neurons and shows a very limited memory. On the other side, chemo-transduction, that is mostly mediated by unmyelinated (C type) fibers, generates relatively slow onset responses, characterized by tonic (not phasic) activity, and displays memory capacity (Ardell and Armour 2016). The second hit, which may dramatically enhances the first one also depending on the extension of the ischemic area, is the reduced cardiac output. The subsequent baroreceptor reflex activation, acutely aimed at sustaining cardiac function at the expense of an increased metabolism, may chronically lead to deleterious effects (Ruffinazzi and Dusi 2020). Indeed, the decrease in mean and pulsatile blood pressure (BP) that may follow I/R injury leads to a decreased afferent nerve firing from stretch receptors within the arterial wall (carotid and aortic baroreceptors), a reduced stimulation of the nucleus tractus solitarius, and a reduced inhibition of cardiovascular sympathetic outflow, which is thus increased (Dusi and Ardell 2020). Notably, after a cardiac damage, even when cardiac output is preserved, the autonomic balance shifts towards a sympathetic predominance due to the offset of the baroreflex control by increased afferent pathological signaling from cardiac receptors. In both cases (reduced or preserved cardiac output), carotid baroreflex circuits are not intrinsically malfunctioning (Floras 2009). Accordingly, baroreflex activation therapy (BAT), currently achieved through an invasive device not suitable for the acute setting, is a promising therapeutic option both for HF with reduced ejection fraction (Dusi et al. 2022) and HF with preserved ejection fraction (Salah et al. 2022). The autonomic imbalance occurring during and after I/R injury is accompanied by a morpho-functional remodeling affecting all cardiovascular autonomic neurons and stations, from the intrinsic cardiac nervous system (ICNS), that is the first integrative center involved in the beat to beat regulation of cardiac functions (Giannino et al. 2024), to the intrathoracic extracardiac centers, and up to the higher centers (Ruffinazzi and Dusi 2020). Neuronal remodeling may lead to denervation, nerve sprouting, and sympathetic hyperinnervation (D’Elia et al. 2014). The dramatic pro-arrhytmic potential of this autonomic imbalance and remodeling is well charactherized at the pre-clinical (Weperen et al. 2023) and clinical level (Dusi et al. 2019; Dusi et al. 2023; Dusi, et al. 2024; Savastano, et al. 2024; Iannaccone et al. 2022). Notably, in the setting of high-frequency and prolonged sympathetic activation such as during acute myocardial ischemia, additional sympathetic co-transmitters other than norepinephrine (NE) are co-released from sympathetic post-ganglionic efferent fibers, including ATP, galanin and neuropeptide Y (NPY) (Herring 2015). NPY is of particular interest because, beyond having the direct effect of reducing ventricular electrical stability through Y1 receptors (Dusi et al. 2020; Kalla et al. 2020), also exerts a potent vasoconstrictor effect thought Y1 (Herring et al. 2019) and Y5 type (Malmström 2002) receptors on coronary micro-arteries and its levels after reperfusion were associated with microvascular dysfunction and poor prognosis both in animals (Herring et al. 2019) and in humans (Herring et al. 2019).

Overall, there is a strong rationale for neuromodulation strategies, aimed at reducing sympathetic activity and/or increasing vagal tone, in the setting of I/R injury. Of note, since this autonomic inbalance is driven and maintaned by an abnormal afferent signaling that affects both sympathetic (Dusi and Ardell 2020) and parasympathetic (Weperen and Vaseghi 2023) fibers, several studies have been conducted to elucidate and modify afferent transmission during I/R. For instance, it was recently demonstrated in a porcine model (Yoshie, et al. 2020) that cardiac afferent signaling mediated by the transient receptor potential cation subfamily V member 1 (TRPV1) channels promotes cardio-cardiac spinal reflexes after MI that result in sympathoexcitation and arrhythmogenic ventricular remodeling. Accordingly, the selective epidural administration of resiniferatoxin, an ultrapotent TRPV1 desensitizers, to DRGs provides cardioprotection against ventricular arrhythmias in pigs by inhibiting afferent neurotransmission during I/R injury (Yamaguchi et al. 2023).

### The neuroimmune crosstalk

The autonomic nervous system and the immune system are tightly related one to each other. Indeed, stimulation of adrenergic receptors on immune cells controls all crucial stages of immune responses (Dusi 2020) and sympathetic overactivity was associated with the development and exacerbation of several chronic immune‐mediated diseases (Bellinger and Lorton 2014). On the other hand, the parasympathetic nervous system acts as a powerful anti‐inflammatory neural circuit, both at the central and at the peripheral level. As for most neuronal pathways, the afferent branch plays a pivotal role in this process. The activation of afferent vagal fibres, typically elicited by inflammatory cytokines and ROS, but also by other kinds of chemical or mechanical stressors, stimulates the NTS of the medulla oblongata with several consequences (Trakhtenberg and Goldberg 2011). On one side, NTS activation reflexively elicits nearby corticotrophin-releasing factor (CRF)-releasing neurons (Chen et al. 2021). Such stimulation activates the hypothalamic-pituitary-adrenal system, with release first of ACTH and finally of glucocorticoids, with potent anti-inflammatory activity. On the other side, after subcortical processing, NTS activation leads to an increase in vagal output that in turn suppress peripheral inflammation, thus completing the inflammatory reflex mechanism better known as cholinergic anti-inflammatory pathway (CAP). Notably, recent data suggest that pro- and anti-inflammatory cytokines interact with distinct populations of vagal neurons within the NTS to alert the brain of an emerging inflammatory response (Jin et al. 2024). Genetic manipulation of these neurons was proved to profoundly affect inflammatory responses, therefore opening new possibility of neuroimmunomodulation (Jin et al. 2024).

**Fig. 1:**
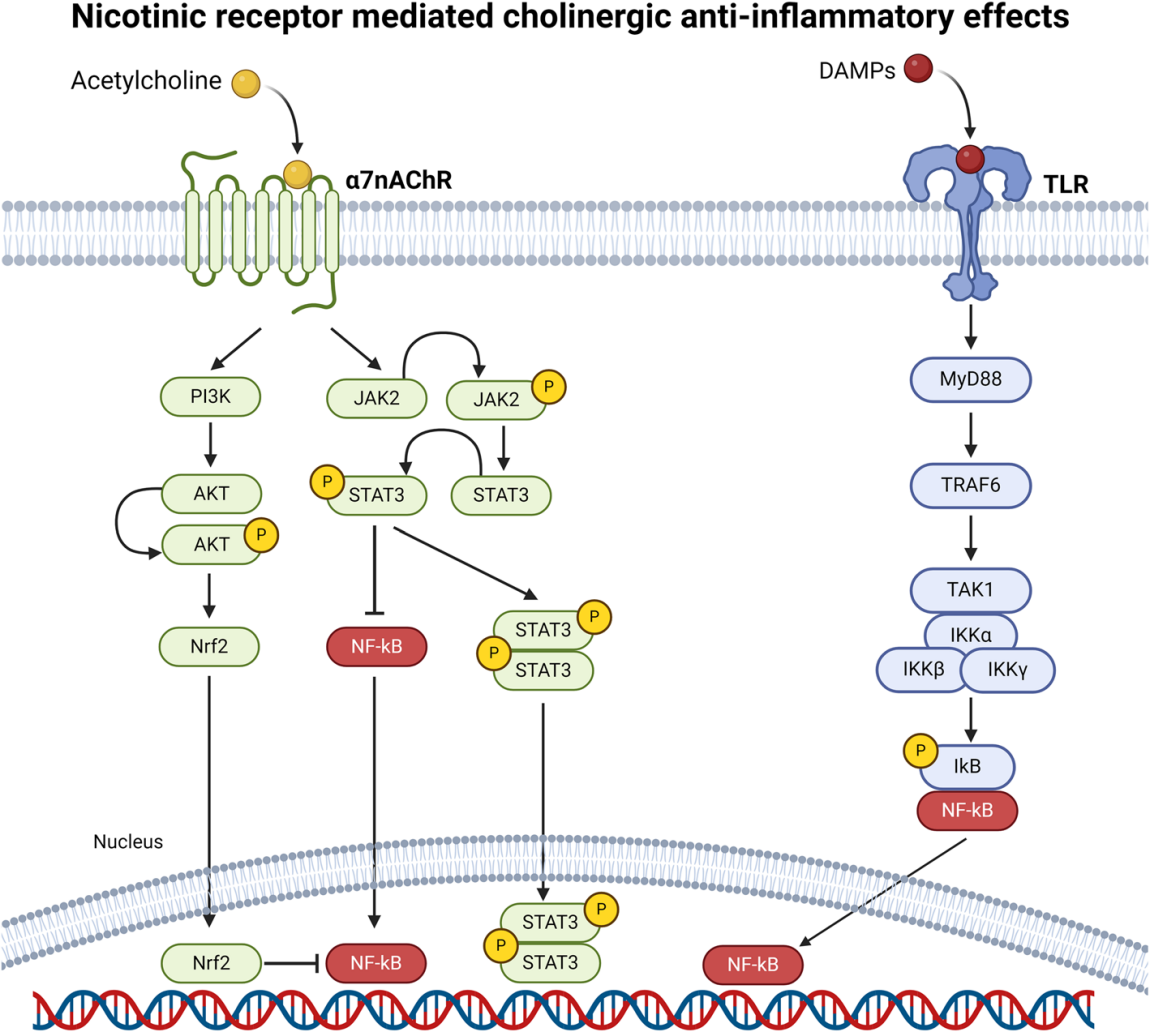
Nicotinic receptor mediated cholinergic anti-inflammatory effects. Acetylcholine (ACh, in green in the figure), released by parasympathetic post ganglionic terminals and, in the spleen, by ACh- producing T-cells (vagal-splenic pathway, details in the text), binds to nicotine acetylcholine receptors (a7nAChR), a ligand-gated ion channel protein, on monocytes/macrophages surface. This binding plays a key role in mediating cholinergic anti-inflammation effects, by activating phosphatidylinsotisol 3-kinase (PI3K) with Nrf2 (later effector of this molecular pathway) and Janus kinase/signal transducer and activator of transcription (JAK2/STAT3) pathway, a downstream intracellular mechanism leading to the inhibition of Nuclear Factor kappa B (NF-kB) nuclear translocation, resulting in suppression of pro-inflammatory cytokines expression and production. In parallel, during immune/inflammatory processes (such as I/R injury) the innate response is triggered by the release of DAMPs (in red in the figure), predominantly through TLR2 and TLR4, broadly expressed in the cardiac tissue. After activation, MyD88-signalling pathways results in NF-kB activation, with production of pro-inflammatory cytokines. Created with Biorender.com. Alfa7nAChR: alfa-7-acetylcholine receptor; DAMPs: damage-associated molecular patterns; IKK: IkappaB kinase; JAK2: Janus kinase 2; MyD88: Myeloid differentiation primary response 88; NF-kB nuclear factor kappa-light-chain-enhancer of activated B cells; Nrf2: factor erythroid 2–related factor 2; PI3K: phosphatidyl-inositol 3-kinase; STAT3: Signal transducer and activator of transcription 3; TLR: Toll-like receptors; TRAF6: TNF receptor associated factor 6

The CAP, first proposed by Tracey (Tracey 2002), is a peripheral reflex that consists in the direct inhibition of pro-inflammatory cytokine release (TNFα, IL-1β, IL-6, IL-18) from local macrophages and other immune cells induced by the neuronally released ACh. The molecular mechanism is the binding of ACh to the α7 subunit of nicotinic receptors (α7nAChR) (Tracey 2007). Therefore, cholinergic neurons can reflexively regulate inflammatory responses in real time (within seconds). The spleen is the only visceral site not directly affected by this efferent vagal reflex (Bellinger et al. 1993). Indeed, post ganglionic splenic neurons originating in the celiac ganglion are adrenergic, not cholinergic (Pereira and Leite 2016; Rosas-Ballina et al. 2011) , and the release of norepinephrine subsequently stimulates ACh- producing T-cells (Andersson and Tracey 2012); this process is named vagal-splenic pathway (Davidson et al. 2020). Figure 1 summarizes the molecular pathways underlying the nicotinic receptor mediated cholinergic anti-inflammatory effects. In addition to these nicotinic mediated effects, ACh improves mitochondrial function by reducing mPTP opening (Miao et al. 2013), inhibits programmed cell death and promotes mitochrondriogenesis (Lu et al. 2013). Overall, vagal activation on the one hand directly and indirectly reduces the inflammatory burden and on the other hand reduces ROS production, interrupting the vicious cycle of ischemia-ROS production-inflammation-ROS release.

In 2011 Calvillo et al (2011) demonstrated the cardiac effects of this pathway, underlying the crucial role played by α7nAChR in the HR- independent anti-inflammatory and anti-apoptotic effect of cVNS. Indeed, right cVNS, applied beginning 5 minutes before myocardial ischemia till 5 minutes after reperfusion (for a total duration of 40 minutes) in anesthetized rats, was associated with a decrease in infarct size and inflammatory markers independently of the heart rate (HR). At the cardiac level, the strong and direct anti-inflammatory effect of vagal activity is a key component of sympathetic-parasympathetic antagonism. Figure 1 shows the intracellular effect of α7nAChR.

Of note, the neuroimmune crosstalk is much more complex than anticipated, including also intracellular pathways and modulatory effects, such as beta-adrenergic receptor intracellular desensitization in immune cells. Indeed, Moragon et al (2020) recently demonstrated in a mice ischemia/reperfusion model (45 min ischemia/24 hours reperfusion) that metoprolol, but not atenolol or propranolol, significantly attenuated neutrophil infiltration and ameliorated I/R injury. Different intracellular β1 adrenergic receptor conformational changes were described by the in-silico analyses following metoprolol bounding as compared to the other two β-blockers and advocated as potential explanation for the different responses. These effects are expected to be synergic to those of VNS, as already demonstrated in the setting of HF (Hamann et al. 2013).

## Principles of electrical vagal modulation

### The target: Anatomy of the parasympathetic branch of the ANS

The efferent parasympathetic branch of the autonomic nervous system distinguishes, in the traditional two-neurons model, a preganglionic neuron, whose soma is located at the level of the dorsal motor nucleus of the vagus and the nucleus ambiguous, both within the medulla oblongata, and a postganglionic neuron (Dusi and Ardell 2020). Post ganglionic neurons are located directly on the posterior epicardial surface of the atria and superior surface of the ventricles, where they form, together with afferents neurons, local circuit neurons and a small number of catecholaminergic-releasing neurons, the intrinsic cardiac ganglionated plexi, also referred to as the intrinsic cardiac nervous system (ICNS) (Giannino et al. 2024). The axons of cardiac pre-ganglionic neurons travel along the vagus nerve (VN), together with other non-cardiac pre-ganglionic neurons directed towards upper and lower respiratory organs, gastrointestinal organs, and ovaries, and afferent neurons, with afferent fibres being much more abundant than the efferent ones (80% versus 20%). VN fibers, classified according to the diameter and the conduction velocity, range from Aα, the largest and fastest, to unmyelinated C-fibers (Mei et al. 1980), the smallest and slowest with highest activation thresholds (Schwaber and Cohen 1978; Jones et al. 1995). Cardiac vagal control in mammalians relies on B-type fibers (McAllen and Spyer 1976) (efferent fibers, with neuronal bodies mostly located within the nucleus ambiguous (Hopkins and Armour 1998) and projecting to the sinus node (Geis and Wurster 1980)) and C-type fibers (afferent and efferent, with the neuronal bodies of the efferent ones mostly located within the dorsal motor nucleus of the vagus (Cheng et al. 1999) and projecting to the the ventricles (Geis and Wurster 1980; Gourine et al. 2016)). The distribution of pre-ganglionic fibers to the post-ganglionic neurons within the epicardial fat pads is asymmetric: the right VN has a larger influence on the sinus node activity, whereas the left VN has a predominant control over the atrio-ventricular node function (Zandstra 2021). Both directly affect atrial and ventricular cardiomyocytes (Zandstra 2021). Indeed, homogenous data from the literature confirms the presence of a direct vagal effect on ventricular electrical and mechanical activity which is independent from the vagal effects on HR (Dusi and Ardell 2020). Accordingly, as opposed to the classical, old conception that cardiac vagal control only affects atrial and nodal tissues, acetylcholinesterase (AChE) staining in animals (Coote 2013) and humans (Kawano et al. 2003) clearly showed that cardiac cholinergic innervation is widespread across all cardiac chambers. Finally, cholinergic muscarinic receptors were localized in both the right and the left ventricle (Coote 2013; Fields et al. 1978). Notably, very relevant for vagal neuromodulation in pathological conditions, Vaseghi et al (2017) demonstrated in a porcine model the anatomical integrity of the cardiac parasympathetic neuronal network in border zones and in viable myocardium of infarcted hearts (as opposed to the sympathetic fibers). Despite anatomically intact, postganglionic parasympathetic neurons, studied with in vivo neuronal recordings, showed abnormal functionality both at rest and in response to stimuli, proving a complete disruption of cardiac vagal control after MI and reinforcing the strong rationale for therapeutic interventions aimed to restore a proper cardiac vagal output.

The main neurotransmitter released from the parasympathetic postganglionic terminal is acetylcholine (ACh), that exerts its action through binding to M1-M5 muscarinic metabotropic receptors (Finlay et al. 2017). Additional parasympathetic cotransmitters include NO, that facilitates ACh release and inhibits that of NE (from sympathetic neurons) at the presynaptic level, and VIP (vasoactive intestinal peptide), which may directly affect the coronary tone with vasodilatory effects, as well as the myocardium (Feliciano and Henning 1998; Henning and Sawmiller 2001). M2 receptors, a Gi-coupled type of receptors, are the most represented at the cardiac level and their activation reduces cAMP concentration and activates the G protein-coupled inwardly rectifying potassium channels (GIRK) channels (Bywater et al. 1989), being responsible for the chronotropic, dromotropic, lusitropic, inotropic, and bathmotropic negative effects of vagal stimulation (Giannino et al. 2024). GIRKs, better known as IKACh, are the main responsible for the short-term onset strong negative chronotropic effect of vagal activation. From a purely electrical point of view, the antiarrhythmic effect of efferent vagal activation at the ventricular level mostly relies on the antagonism of the pro-arrhythmic effect of excessive sympathoexcitation and the consequent intracellular calcium overload (Dusi et al. 2020).

**Fig. 2:**
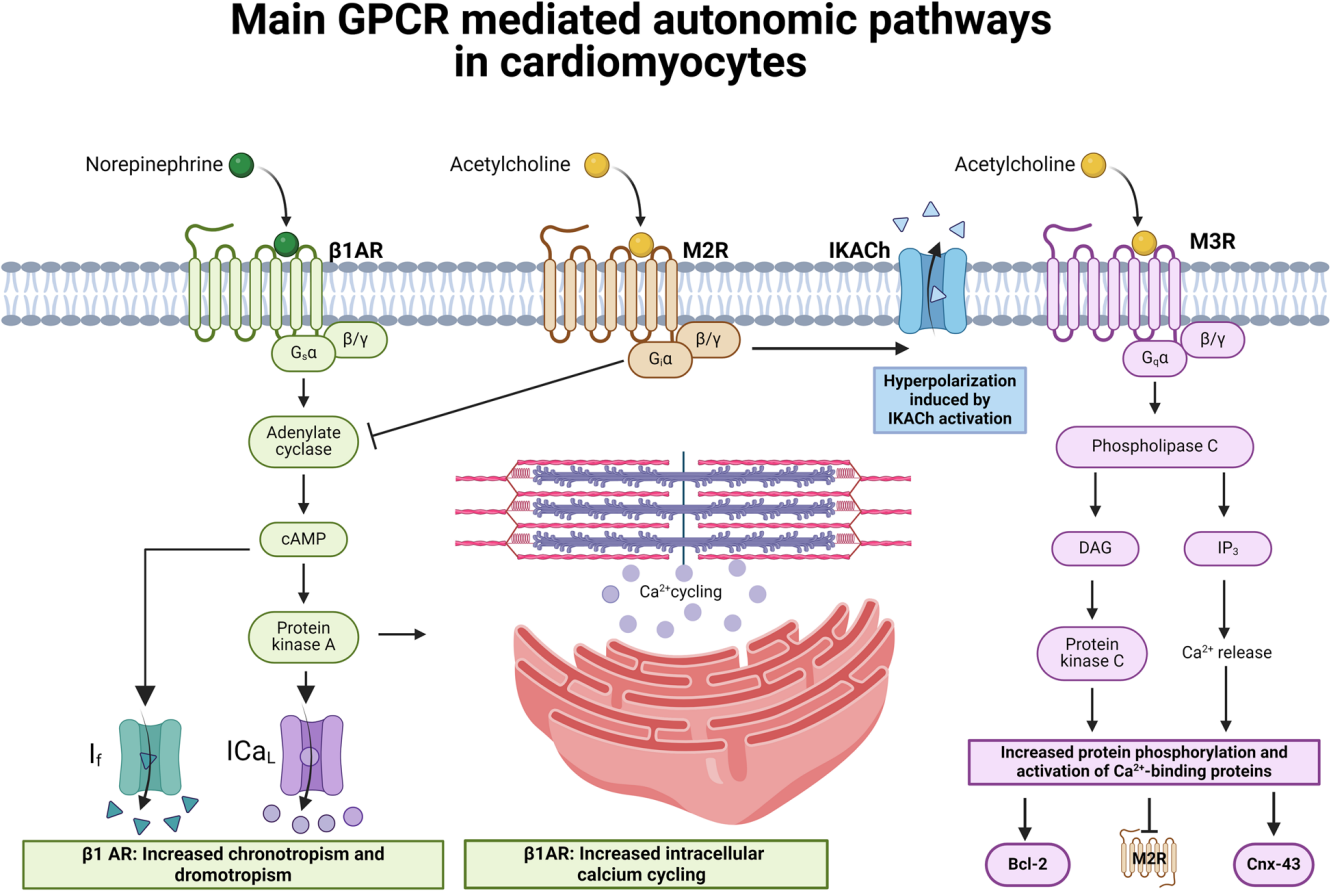
Main GPCR mediated autonomic pathways in cardiomyocytes. The main neurotransmitter released by the vagus nerve is acetylcholine (in yellow in the figure), that exerts its cardiac effects mostly through M2 receptors, and, to a lesser extent, through M3 receptors. M2 receptors are a Gi-coupled type of receptors that inhibit adenylate cyclase and the production of cyclic adenosine monophosphate (cAMP), the main second messenger of β1-adrenergic receptors. Additionally, M2 receptors are also coupled with G protein-coupled inwardly rectifying potassium channels (GIRKs), better known as IKACh, that are the main responsible for the short-term onset strong negative chronotropic effect of vagal activation. Overall, M2 receptors are responsible for the chronotropic, dromotropic, lusitropic, inotropic, and bathmotropic negative effects of vagal activation. In the setting of I/R injury, they their activation antagonizes at the intracellular level the effects of catecholamines and protects the cells from intracellular calcium overload. In parallel, acetylcholine binding to M3 receptors promotes the release of diacylglycerol (DAG) and inositol-3-phosphate (IP3), which are instead involved in the negative modulation of M2 receptors, as well as in the activation of survival and cytoprotective pathways (Bcl-2; Connexin-43). β2 adrenergic receptors (a Gi-coupled type of receptors) are not shown for simplicity. Created with Biorender.com. β1AR: β1-adrenergic receptors; Bcl-2: B-cell lymphoma 2; cAMP: cyclic adenosine monophosphate; Cxn-43: connexin-43; DAG: diacylglycerol; ICaL/T: If: funny current; IP3: inositol-3-phosphate; MR: muscarinic receptor

On the other hand, M1 and M3 receptors, which are also expressed in the heart to a much lesser extent, are coupled to Gq/11 proteins and elicit the PLC pathway and therefore the production of inositol 3-phosphate (IP3) and diacylglycerol (DAG), which may act as feedback counter-regulatory actions to M2 receptor activity (Saternos et al. 2018). For instance, Tsuchida et al (Tsuchida et al. 2013) suggested that the positive inotropic action described after the direct exposure to high dosages of ACh in isolated human (Du et al. 1995) as well as animal cardiomyocytes (Gilmour and Zipes 1985; Yang et al. 1999) as opposed to the M2 receptor mediated negative inotropic effect of neuronally released ACh, may be mediated by the stimulation of M3 receptors. M3 receptors also play a cardioprotective role in models of myocardial infarction by activating several cytoprotective molecules, such as Bcl2, and cell adhesion or communication structures, such as connexin-43, in order to increase survival signals (Yang et al. 2005). Finally, the expression and the role of M4 and M5 receptor at the cardiac level is still debated, but there is no definitive prove so far (Palma 2024). Figure 2 summarizes the main G protein coupled receptors (GPCR) mediated autonomic pathways in cardiomyocytes.

Concerning the fibers’ spatial organization within the VN trunk at the cervical level, most pre-clinical data suggest an organotopic and function-specific distribution (Settell et al. 2020). Thompson et al (Thompson, et al. 2024), using an elegant combination of trial-and-error selective VNS and *ex vivo* micro-computed tomography fascicle tracing, recently showed in a porcine model a significant spatial separation of cardiac afferent and efferent fibers at the mid-cervical level. They also showed that cardiac afferent fibers are in proximity to pulmonary fibers consistent with recent findings of cardiopulmonary convergent neurons and circuits (Devarajan et al. 2022). According to most anatomical studies in humans, the auricular branch of the VN (ABVN) is the afferent cutaneous branch which innervates the antihelix, the tragus and the cavity of the concha (Peuker and Filler 2002). Interestingly, as early as 475-221 Before Christ, the healing power of the auricle was included in the Nei Jing, an ancient Chinese text. In Chinese ear acupuncture, the region affecting the heart was located precisely at the tragus level (Wirz-Ridolfi 2019). Accordingly, Frangos et al (2015) recently confirmed through functional MRI that stimulating the auricular branch of the VN in humans at the cymba conchae level activates the same brain regions as cervical VNS.

### The devices: bioelectronic medicine applied to parasympathetic modulation

The expression “Bioelectronic Medicine” refers to an electrical current delivered to a nervous tissue (central or peripheral) to achieve targeted therapeutic benefits. Although conceptually the first use dates to the time of ancient Greeks and Romans, who used electric shock from torpedoes rays to relieve pain, it was not until the 19^th^ century that bioelectronic studies began to take hold in routine clinical practice. However, in the absence of strong evidence, they were gradually abandoned in favour of drugs, until the last 30 years, when a renewed interest on electrical neuromodulation has emerged for several drug-refractory conditions (Heidland et al. 2013). In this context, vagus nerve stimulation at the cervical level (cVNS), where the nerve can be easily accessed surgically, has become increasingly popular. Due the extensive nerve distribution of the VN to various organs, cVNS has the potential on one side, to modulate a plethora of responses, on the other side, depending on its characteristics, to elicit off-target side effects. Since its first human approval by FDA in 1997 for drug-refractory epilepsy, to date studies on cVNS range from modulation of autoimmune, gastrointestinal, infectious, and cardiologic diseases (Fang 2023).

The two essential components of an electrical neuromodulation system are the generator of the electrical current and the electrode (or the electrodes) that delivers the current to the target. Based on the location of the entire system, electrical neuromodulation strategies can be classified in invasive in case of a cruel implantation or non-invasive in case of an entirely percutaneous systems. Based on the type of fibres targeted, the stimulation can be either selective or not selective. Finally, based on the possibility of integrating a biofeedback from the targeted organ/system by the stimulator, the stimulation can be at open-loop (no biofeedback) or at closed loop (with biofeedback).

In addition to the classical way of achieving VNS, namely invasive cervical VNS thought an electrode implanted in the neck around the VN, in the last decades additional non-invasive techniques have been evaluated, including cervical non-invasive VNS (nVNS) and transcutaneous auricular VNS (taVNS). Cervical nVNS has been only evaluated for neurological disorders (migraine (Fernández-Hernando et al. 2023) and acute cluster headache (Fernández-Hernando et al. 2023)) so far, with an an outstanding tolerance and safety profile and promising efficacy results. The GammaCore (gammaCore®, electroCore, Inc.) is the only FDA approved clinical device for nVNS, in which the two electrodes are embedded on top of the generator. It is not very suitable for the usage in critical patients, where the neck region might be needed for other vascular/invasive procedures. On the other side, taVNS beyond being a not expensive technique, almost free from infectious risk, is also suitable for an acute setting since stimulation is applied at the ear, far from sites that might be used for interventional purposes. TaVNS is being investigated for a wide range of clinical applications but is still not FDA-cleared for any of them.

The first two most widely used commercially available taVNS devices were the NEMOS (NEMOS, taVNS technologies) and the Parasym (Parasym Ltd). The NEMOS device has been granted the European mark (CE certification, indicating legal conformity and safety, but not necessarily clinical efficacy) for the treatment of resistant epilepsy and depression in 2010, for chronic pain in 2012 and for anxiety in 2019 (Farmer et al. 2020). The second-generation device produced by the same company as the NEMOS, named Tvns-L, has CE mark since 2021 for several indications including neurological (anxiety, autism, cognitive impairment, depression, epilepsy, migraine, Parkinson's, Prader-Willi Syndrome, sleeping disorders, stroke, tinnitus), cardiological (atrial fibrillation) and immune/inflammatory (asthma, Crohn's disease, fibromyalgia) conditions. The first generation (Parasym) and the second-generation (Nurosym) taVNS device from the Parasym Ltd company have both been granted CE mark. The main differences between these systems are in the characteristics of the stimulating electrode, that only in the Parasym device has a clip design. The Parasym device has been the most studied for cardiovascular disorders so far, with one randomized study in atrial fibrillation (Stavrakis et al. 2020), two randomized studies in HF with preserved EF (Stavrakis et al. 2022; Tran et al. 2019), one randomized study in acute decompensated HF (Dasari, et al. 2023) and one randomized study in postural tachycardia syndrome (Stavrakis et al. 2024).

An additional device, the taVNS Stimulator by Soterix Medical, is currently also available, and it’s the only one with 3 different types of electrodes available (including a clip-type).

Concerning the safety profile, the non-invasive nature of taVNS granted it an impressive safety record. A systematic review by Redgrave et al (2018) summarized all the available literature on taVNS, not limited to cardiovascular diseases, and found that local irritation (tingling/pain/redness/itching) is the most common discomfort (16.7%) that is attributable to stimulation, with headache being the second (3.3%) and nasopharyngitis (1.6%) the third. Dizziness and syncope had a prevalence of 1.4%. However, only three severe adverse events were considered taVNS-related.

## The dose response issue

Unlike drugs, electrical neuromodulation requires, to obtain a single "dose" of treatment, the integration of dozens of different parameters, which depend both on the device-target interface and on the mode of energy delivery, so that the chosen "dose" of treatment can vary among hundreds of possibilities (Dusi et al. 2022). Table 1 summarizes the most relevant, that can be academically divided into electrode and current-related parameters, stimulation modality–related parameters, and safety parameters.

**Table 1:** Parameters that can be modified in the setting of electrical neuromodulation. Modified from De Ferrari 2014

**Electrodes and current related parameters**	**Stimulation modalities related parameters**	**For closed-loop systems: safety parameters**
Electrode and waveform configuration	Right vs left vs bilateral stimulation	Limits for stimulation withdrawal (e.g. low heart rate)
Pulse amplitude, pulse frequency, and duty cycle (duration of the ON/OFF cycles)	In case of a neuronal structure containing both afferent and efferent fibers: bidirectional efferent and afferent (technically easier) vs preferential efferent or preferential afferent stimulation (technically more complex)	Type of biofeedback used
	Selective versus non-selective stimulation	
	Continuous stimulation versus respiratory and/or pulse-synchronous stimulation	
	With pulse-synchronous stimulation: delay from the R wave (or other trigger) and number of pulses per cycle	
	Open loop versus closed loop stimulation	
	Titration protocols	

Regarding the interface, depending in fact on the material used, its geometry, the location of the electrode, the number of poles, and how it relates to the organ, there might be different resistances offered to the passage of current (González-González et al. 2024); on the other hand, as far as the mode of energy delivery is concerned, regardless of whether a biofeedback is integrated or not, there are many modulable parameters, such as pulse frequency, pulse width, on/off cycle duration (duty cycle), and active waveform; or again, for wave amplitude, current amplitude and titration method used to reach that amplitude (Elamin et al. 2023).

Such complexity reflects the highly integrated and extremely dynamic behavior of the therapeutic target, namely the ANS, that is still far from being fully understood.

For cVNS, a major issue, that is deeply influenced by the stimulation parameters and the electrode’s characteristics, is represented by the choice of the type of stimulation to pursue to improve efficacy and limit/avoid off target side-effects, namely a preferentially efferent stimulation rather than a combined afferent and efferent stimulation and, even better, an organ/fibres specific stimulation (otherwise known as selective stimulation). The main limit to selective cVNS is the still limited knowledge of the functional anatomical organization of the vagus nerve across species and particularly in humans (Ottaviani and Macefield 2022). Transauricular VNS is by definition a purely afferent kind of VNS, yet the stimulation protocol may have a profound impact on the central effects and the consequent modulation of efferent vagal output (Farmer et al. 2020). For instance, most clinical applications of taVNS used a 20–30 Hz frequency stimulation, but Sclocco et al (2020) reported a significantly greater effect of high-frequency (100 Hz) stimulation on the modulation of medullary vagal nuclei activity. Also, another critical issue is whether taVNS should be delivered in a respiratory gated way. Indeed, physiologically, afferent inputs to the nucleus tractus solitarius (NTS) are modulated by rhythmical oscillations in the respiratory cycle, with a major degree of receptivity during exhalation (Gilbey et al. 1984; Paton et al. 2022). Accordingly, gating taVNS to exhalation was proved to significantly potentiate its effects on vagal medullary nuclei activity, peripheral cardiovagal response and blood pressure levels (Sclocco et al. 2019). This technique is known as respiratory-gated auricular vagal afferent nerve stimulation (RAVANS) (Garcia et al. 2022).

Unfortunately, clinical trials were started long before the dose issues were solved, resulting in different and often not comparable stimulation protocols, that may contribute to understand the translational troubles encountered, for examples, for cVNS in the setting of chronic HF, where most randomized clinical trials were unable to fully confirm the promising preclinical results (Dusi and Ferrari 2021; Ferrari 2014).

## Preclinical data on cervical VNS

### Arrhythmias and heart failure

The beneficial effects of invasive cVNS have been studied for decades in pre-clinical models of acute and chronic myocardial ischemia, mostly focusing, in the beginning, on the acute antiarrhythmic efficacy and on the chronic anti-remodelling benefits, and only more lately directly assessing the acute effects on I/R injury.

Between 1973 and 1978, several studies on anesthetized animals (Yoon et al. 1977; Kolman et al. 1975; Kent et al. 1973; Myers et al. 1974) showed that cVNS reduces the risk of VF during acute myocardial ischemia; one of them (Myers et al. 1974) also demonstrated that the acute antifibrillatory effect of cVNS was not abolished by preventing HR decrease nor by VN decentralization, underscoring a direct ventricular efferent effect. The final demonstration came in 1991 from a conscious canine model of sudden death (Vanoli et al. 1991): cholinergic antagonism favored VF, while right cVNS, applied a few seconds after a 2-minutes circumflex coronary occlusion in the setting of a previous anterior MI and an ongoing exercise stress test, protected against VF. Almost 50% of the antifibrillatory effect was due to the significant right cVNS induced HR lowering.

Starting from the early years 2000, right cVNS began to be evaluated in models of chronic HF with reduced ejection fraction (HFrEF). In 2004, Li et al (2004) showed in a randomized study on conscious rats with a previous 14 days old large anterior myocardial infarction, that right cVNS stimulation applied for 6 weeks was associated with better left ventricular (LV) function, lower normalized biventricular weight, lower norepinephrine and BNP levels and a strikingly better survival (86% versus 50%) at 140 days compared to sham-operated animals, despite a similar infarct size. Right cVNS (10 sec on, 50 sec off) was delivered at 20 Hz, with 0.2 ms pulses and the intensity was adjusted to reduce HR by 20-30 bpm from a starting value of 360 bpm. The beneficial effects of right cVNS (set to achieve a 10% HR lowering effect) were subsequently confirmed on a canine model of coronary microembolizations induced HFrEF (Sabbah et al. 2011) and proved to be additional to those achieved with metoprolol alone (Hamann et al. 2013). In addition to the favorable LV remodeling, a significant impact of a 3-month period of cVNS on several biomarkers was also demonstrated: TNF-α and IL-6 decreased, while NOS and connexin 43 (Cx43) expression tended to normalize. cVNS still retained beneficial effects when applied to the same animal model using a different VNS stimulation protocol specifically set with a low stimulation intensity, unable to affect HR (Hamann et al. 2013). Accordingly, VNS elicited significant benefits in LV hemodynamics, C-reactive protein, norepinephrine and angiotensin II levels, HR variability and baroreflex sensitivity, compared to controls, even in a high-rate ventricular pacing induced HFrEF canine model (Zhang et al. 2009). VNS intensity was individually set before starting pacing to reduce sinus rate by approximately 20%, but HR was subsequently kept constant by pacing. These data were recently confirmed by an elegant optogenetic study (Machhada et al. 2020) showing that a purely efferent VNS with almost no impact on HR led to a significantly better cardiac function (compared with the corresponding sham-control group) both in normal and in post-MI rats, despite no changes in myocardial infarct size (30% of LV mass). VNS was achieved through a 4-week program of intermittent optogenetic activation of cholinergic neurons in the vagal dorsal motor nucleus, and, in the infarcted animals, it was started 48 hours after MI induction, therefore not leading to an infarct size reduction, that would have required an earlier application. Impressively, LV function almost normalized in post-MI rats, despite a similar infarct size (at least 30% of LV mass), an effect much more pronounced than the one observed in the landmark model by Li et al (2004) , where, as previously mentioned, cVNS was started 14 days after MI. Finally, the authors also reported an improvement in cardiac function caused by optogenetic efferent VNS in normal rats, that was related to a reduced myocardial expression of GRK2 (G protein–coupled receptor kinase 2) and of β-arrestin 2, both involved in β1-adrenergic receptor desensitization and internalization. These data suggest the apparently paradoxical potential for cardiac efferent vagal activation to improve sympathetic responses of cardiomyocytes despite its well established anti-adrenergic effects, underscoring the complexity of sympathetic-parasympathetic interaction (Dusi et al. 2020) and supporting the recent concepts that that cardiac vagal nerve activity actually increases (rather than decreasing as traditionally believed) during exercise, and that the amount of cardiac vagal tone may not only reflect the fitness level of a subject, but may also determine his/her cardiac performance (Gourine and Ackland 2019). Additionally, back in 1998, Feliciano and Henning demonstrated that 5 minutes trains of bilateral cVNS at 20 Hz applied in anesthetized healthy dogs during muscarinic and beta-adrenergic blockade, cause significant coronary artery dilation that is prevented by a VIP antagonist (Feliciano and Henning 1998). A dynamic modulation of coronary artery blood flow during exercise associated with an increase in cardiac vagal activity (directly measured) and mediated by VIP (Shanks et al. 2023) was recently confirmed in a conscious sheep model. The increased NO availability associated with cVNS in animal models (Brack et al. 2007) and proved to be dependent upon the stimulation parameters (higher during low voltage and high frequency stimulation) may also contributes to this effect (Allen et al. 2022).

Concerning the dose response issues, some pre-clinical studies were performed to shed light on this matter. Kong et al (2012) evaluated the efficacy of different protocols of right cVNS, applied for 240 minutes after coronary artery ligation in anesthetized rats, in reducing the infarct size. They found that the combination of parameters leading to the largest cardioprotective effect (lower amplitude, lower frequency, and longer duration of stimulation) was not that producing the greater HR decrease. These data suggest that a purely efferent cVNS, even if feasible, may not necessarily be the best option, since afferent fibers activation might significantly contribute to the beneficial effects of cVNS. Indeed, sensory afferent vagal fibres, whose bodies reside in the nodose ganglion, sense cytokines and metabolites released during inflammation/ischemia, transmitting the signals to upper nervous structures (such as NTS) involved in generating brain-derived neural control of inflammation (Dusi and Ardell 2020; Dusi 2020). Accordingly, back in 1973 (Schwartz et al. 1973) Schwartz et al demonstrated that selective afferent cVNS increases contralateral cardiac vagal output while reducing ipsi- and contralateral cardiac sympathetic output (Schwartz et al. 1973). In order to clarify the relative contribution of afferent versus efferent fibers activation to the acute HR responses elicited by chronic right cVNS, a wide range of stimulation parameters was recently tested in a canine conscious model (Ardell et al. 2017), including electrode configuration (anode/cathode inversion), pulse frequency (2–20Hz), intensity (0–3.5 mA) and pulse widths (130–750*μ*s). HR responses were determined for each combination over 14 months. Based on frequency–amplitude–pulse width, the authors identified an operating point, defined as “neural fulcrum” where a null HR response was evoked during the on-phase of cVNS. They showed that chronic cVNS, when delivered within the constraints of the neural fulcrum in healthy animals, was able to maintain the circadian control of HRV. In 2023 (Hadaya et al. 2023), the same group reported for the first time the effects of right cVNS (5 Hz, 250-μs) delivered with a stimulation amplitude decided according to the neural fulcrum, in a mini-pig model of MI. Three groups were studied: control subjects, untreated MI, and MI + right cVNS, with cVNS started 48 hours after distal LAD embolization. Notably, to guarantee therapeutic levels of cVNS, a 4-week titration period was used before MI; cVNS was then stopped after titration at the time of acute MI and then resumed 48 hours later at the previously determined intensity. The authors reported a pronounced mitigation of the unfavorable functional and structural remodeling in the ischemic border zone, in the stellate ganglia and in the dorsal root ganglia (including both neurons and glial cells), that was accompanied by an improvement in cardiac function, a marked reduction in induced ventricular arrhythmias, and the preservation of normal cardiac sympathetic responses compared with untreated MI. Notably, all these favorable effects of cVNS were observed despite no changes in myocardial infarct size between treated and untreated animals, underscoring the importance of a very early parasympathetic modulation in order to also reduce the infarct size (Dusi et al. 2023). The beneficial effects of cVNS on the unfavorable structural remodeling affecting the stellate ganglia fully confirms previous findings showing that continuous low-level cVNS reduces stellate ganglion nerve activity in healthy dogs (Shen et al. 2011).

### Myocardial I/R injury

The first, pioneer demonstration of the protective and only partially HR-dependent effect of right cVNS on reperfusion arrhythmias was provided by our group back in 1987 (Zuanetti et al. 1987), but remained neglected for a long time, until it was finally understood that reperfusion-related arrhythmias and myocardial I/R injury share a common pathophysiological pathway that is antagonized by cVNS. In both settings, the cardioprotective effects of VNS (Kakinuma et al. 2005; Uemura et al. 2010; Mioni et al. 2005), similarly to the ones of ischemic preconditioning (Krieg et al. 2004), are mediated by the intracellular activation of phosphatidylinositol 3-kinase and Akt pathways in cardiomyocytes. The consequence is the upregulation of the anti-apoptotic protein BCL-2 and the suppression of caspase-3, that both concur to a powerful protection from cell death (Katare et al. 2009). Notably, an increase in cardiac efferent vagal tone is also likely to mediate the beneficial effect of remote ischemic preconditioning (Crimi et al. 2013; Donato et al. 2013).

In 2013, Shinlapawittayatorn et al (2013) were the first to compare the acute cardioprotective effects of intermittent and continuous left-sided cVNS in a porcine model of I/R injury, as assessed at the end of reperfusion. Left cVNS (20 Hz, 3.5 mA, 500 μs pulse width) was applied at the onset of a 1-hour complete left anterior descending artery occlusion (LAD) and throughout the end of a 120-minute period of reperfusion, either intermittently (with recurring cycles of 21-second ON and 30-second OFF) or continuously. In both cases, cVNS significantly reduced infarct size, improved LV function, decreased VF episodes, and attenuated cardiac mitochondrial ROS production, compared with the control group, but the amount of benefit, particularly of infarct size and VF episodes reduction, was greater with intermittent cVNS (approximately 89% infarct size reduction). In a subsequent work on the same model (Shinlapawittayatorn et al. 2014) the group assessed whether left cVNS (3.5 mA, 20 Hz, with continuously recurring cycles of 21-second ON, 30-second OFF) applied late during ischemia (30 minutes after LAD occlusion) or at the onset of reperfusion differentially protected against cardiac I/R injury. They showed that left cVNS started during late ischemia, but not at reperfusion, still retained beneficial effects on electrical stability, infarct size (59% reduction), cardiac function, inflammatory biomarkers, ROS production and phosphorylated Cx43 levels, providing an important translational message. Of note, since all the assessments of efficacy were performed at the end of reperfusion, the study cannot evaluate the potential for late beneficial effects of cVNS.

In 2018 (Nuntaphum et al. 2018) the same group assessed the acute effects of intermittent left cVNS with the abovementioned characteristics, according to the integrity of the ipsilateral and contralateral VN in an identical I/R porcine model. Thirty swine were randomized into five groups: I/R injury with no cVNS and left cVNS with both vagal trunks intact (LC-VNS), with left VN transection (LtVNX, selective left efferent stimulation), with right VN transection (RtVNX) and with atropine pretreatment, respectively. LC-VNS and LtVNX produced a similar and more profound cardioprotective effect (using infarct size as primary endpoint, an 89% reduction for LC-VNS and a 84% for LtVNX was observed) compared to RtVNX (63% reduction), suggesting on one side, a predominant ipsilateral efferent fibers stimulation with this cVNS protocol, on the other side, the importance of a bilateral efferent vagal activation, to achieve the full beneficial effects of cVNS in response to such a powerful proinflammatory and prooxidative stressor as I and R. Accordingly, in an in vivo rat model of acute myocardial I/R injury (Wang et al. 2015), the cardioprotective effects of cVNS applied as perconditioning (during ischemia) in combination with limb remote ischemic perconditioning (that was also proved to elicit an increase in cardiac vagal output (Mastitskaya et al. 2012)) were superior to those achieved with either treatment alone, underscoring once again the importance of powerful vagal responses to counteract I/R injury. Notably, in the 2018 study (Nuntaphum et al. 2018) all VNS-treated groups showed a significant reduction in myocardial infarct size and the effect was reversed by atropine. The study also confirmed that cVNS improves mitochondrial function and shifts cardiac fatty acid metabolism toward beta oxidation. An impressive antiarrhythmic effect during I/R was also demonstrated, as indicated by a reduced premature ventricular complexes (PVC) burden, VT/VF incidence, T-wave peak to end and T-wave peak to end per QT interval ratio, suggesting that cVNS decreases the heterogeneity of ventricular repolarization. These effects were related to a combination of reduced myocardial infarct size and increased phosphorylation of Cx43, a key protein in gap junctions that enables intercellular communication and electrical coupling. Accordingly, a reduced Cx 43 concentration at the ventricular level during ischemia has been correlated to a higher incidence of lethal VAs (Lerner et al. 2000).

Another study conducted by Ando et al (2005) further stressed the influence of Cx43 on ventricular arrhythmias during acute myocardial ischemia, as well as the protective effects of VNS. The occurrence of arrhythmias was lower in rats subjected to cVNS, which also showed higher levels of phosphorylated Cx43 when compared to the sham group. The biomolecular pathway through which cVNS may prevent ischemia-induced reduction of phosphorylated Cx 43 is mediated by the inhibition of its degradation through the muscarinic receptor, displaying a complementary beneficial effect compared to the anti-inflammatory one mediated by nicotine receptors. All this evidence suggests that functional preservation of phosphorylated Cx43 by cVNS would play an important antiarrhythmic effect in the setting of acute ischemia.

Finally, a recent pooled review (Xu et al. 2023) including a total of 10 preclinical studies published from 2011 and 2019, assessing the acute cardioprotective effects of cVNS against myocardial I/R injury and satisfying the inclusion criteria, confirmed that cVNS was associated with a significant reduction in myocardial infarct size [Weighted mean difference (WMD): 25.24, 95% confidence interval (CI): 32.24 to 18.23, *p* < 0.001] when compared to the control group. A significant improvement in LVEF (WMD: 10.12, 95% CI: 4.28; 15.97, *p* = 0.001) and end-diastolic pressure (EDP) (WMD: 5.79, 95% CI: 9.84; -1.74, *p* = 0.005) was also detected. Notably, no significant influences of pre-specified covariates (i.e., stimulation type or site, VNS duration, condition, and species) on the primary estimates were identified at the meta-regression.

### Post-cardiac arrest syndrome

A peculiar clinical condition that shares several pathophysiological pathways with I/R injury is the so called post-cardiac arrest syndrome (PCAS). The term refers to the severe condition that may follow a cardiac arrest (CA), whatever the causes are. The syndrome is characterized by myocardial dysfunction, microcirculatory dysfunction, global brain injury, increased vulnerability to infection, and persistent advancing pathology (Penketh and Nolan 2023). Notably, VF complicating an acute coronary occlusion represents the most common cause of cardiac arrest in the adult population in western countries (Fox et al. 2004), further stressing the potential link between PCAS and cardiac I/R injury sometimes as two sides of the same, life-threatening, coin. Accordingly, a reduced vagal tone and an increased sympathetic output were both proved to favor the occurrence of VF during acute coronary occlusion both at the pre-clinical (Schwartz et al. 1984) and at the clinical level (Ferrari et al. 2020).Notably, we were the first to demonstrate that physical inactivity, that has been consistently associated with reduced vagal tone and reflexes (Gourine and Ackland 2019), significantly increases the risk of VF occurring during a first myocardial infarction in humans (Ferrari et al. 2019). Yet, the possibility that interventional neuromodulation strategies, and specifically cVNS may exert acute beneficial effects even on PCAS was only recently unraveled.

Three studies assessed the effects of cVNS in this setting, using a model of asphyxia cardiac arrest in 2 cases (both receiving left cVNS) (Kim et al. 2022; Choudhary et al. 2022) , and one of VF-induced CA in the third one (treated with right c-VNS) (Sun et al. 2018). In the first study (Choudhary et al. 2022), anesthetized rats were assigned to either post-resuscitation cVNS for 2 h or no cVNS (control group) after 12 minutes of CA. In comparison to the control group, cVNS significantly improved 72 h survival and brain functional recovery, reduced the number of damaged neurons in the CA1 hippocampal region of the brain, as well as the levels of plasma troponin I and creatinine. In the second study (Kim et al. 2022), using anesthetized male rats, cardiopulmonary resuscitation (CPR) was performed 450 seconds after pulseless electrical activity. After the return of spontaneous circulation (ROSC), left cVNS (1 Hz, 1 mA, pulse duration 10 ms) was performed for 3 h in the CA + cVNS group. VNS ameliorated mitochondrial dysfunction in the hippocampus after ROSC and improved neurological outcomes. In the third study (Sun et al. 2018), forty rats were randomized into four groups, and all underwent CPR (*n* = 10 each): CPR alone (control); cVNS during CPR; α7nAChR antagonist with cVNS; α7nAChR agonist without cVNS. VF was induced and untreated for 8 min and defibrillation was attempted after 8 min of CPR. cVNS (10 Hz, voltage 2–6 V, pulse duration 0.5 ms) was initiated at the beginning of precordial chest compressions and continued for 4 h after ROSC. VNS-treated animals showed a better post-resuscitation myocardial and cerebral function and improved survival. The protective effects of cVNS were abolished by the α7nAChR antagonist and reproduced by the α7nAChR agonist, once again confirming the importance of the CAP. In addition, cVNS decreased the number of electrical shocks and the duration of CPR required for stable ROSC.

## Preclinical data on taVNS in I/R injury

Like cVNS, taVNS has been mostly evaluated in preclinical models of chronic HF so far (both with reduced (Wang et al. 2014) and with preserved EF (Zhou et al. 2019)), with promising results. In a rat model of HF with preserved EF induced by high salt intake (Zhou et al. 2019), taVNS, beyond promoting a favourable reverse remodelling with reduced cardiac inflammation and fibrosis, was associated with a lower expression of pro-inflammatory and pro-fibrotic genes (TNF-α, osteopontin, interleukin (IL)-11, IL-18 and IL-23A). Chronic taVNS was delivered at 20 Hz frequency, 2 mA amplitude and 0.2 ms pulse duration, for 30 minutes daily over a 4-week period. Notably, during acute stimulation active taVNS induced a small, but significant heart rate drop. Only a few studies assessed the effectiveness of taVNS in the acute setting of myocardial I/R injury (Elamin et al. 2023).

A combined acute and chronic study was the one reported in 2014 by Wang et (2014), that assessed the long-term effects of low-level tragus stimulation applied since the day of coronary artery ligation in conscious dogs with anterior MI. In the treatment group, taVNS (20 Hz, pulse width 1 ms, with duty cycle of 5 sec on and 5 sec off) was delivered 4 hours per day bilaterally (from 7 to 9 AM and from 4 to 6 PM) to the tragus at 80% below the threshold which slowed sinus rate. After a 90-day period follow-up, taVNS significantly reduced LA and LV dimensions, improved LV systolic and diastolic function and reduced infarct size by ≈50% compared with MI group. These beneficial effects were accompanied by reduced levels of profibrotic factors (collagen I, collagen III, transforming growth factor β1, and matrix metallopeptidase 9), hs-CRP, norepinephrine and NT-proBNP starting from the 7th day to the end of follow-up. In the same canine model (Yu et al. 2016), it was subsequently demonstrated that a right-side only low-level taVNS lasting 2 hours/day per 2 months significantly reduced VAs inducibility, decreased cardiac sympathetic output, as testified by a reduced left stellate ganglion (LSG) activity, and attenuated cardiac sympathetic remodeling induced by chronic MI compared to controls, as testified by NGF downregulation and small conductance calcium-activated potassium channel type2 (SK2) protein up-regulation within the LSG.

In the neurological field, a number of pre-clinical studies indicate that taVNS delivered in acute middle cerebral artery occlusion reduces infarct size through anti-inflammatory effects, reduced excitotoxicity and increased blood-brain barrier integrity (Baig et al. 2022). The long term neuroprotective effects of taVNS in stroke may be mediated by microglial polarization, angiogenesis and neurogenesis (Baig et al. 2022), with peroxisome proliferator-activated receptor-*γ* (PPAR-*γ*) being a candidate mediator of taVNS-induced angiogenesis and neuroprotection against cerebral I/R injury (Li 2020). A recent systematic review and meta-analysis (Melo et al. 2023) including a total of 8 pre-clinical studies published between 2015 and 2022, confirmed, albeit with the limitations of a high heterogeneity and high risk of bias of the included studies, a significant reduction in neurological deficits (SMD = -1.97, 95% CI -2.57 to -1.36, I2 = 44%) and in neuronal infarct size (SMD = -1.51, 95% CI -2.42 to -0.60, I2 = 58%) following taVNS. Markers of neuroplasticity were also significantly improved, as testified by an increased microcapillary density, CD31 proliferation, and brain-derived neurotrophic factor (BDNF) protein levels and RNA expression. This data underscores the strong cytoprotective effect of taVNS in the setting of I/R injury independently on the cell type exposed to ischemia, as also suggested by the favorable pre-clinical results of cVNS in PCAS. Of note, as expected, as for cardio protection, even for neuroprotection, amelioration of mitochondrial dysfunction played a pivotal role (Kim et al. 2022).

## Summary of pre-clinical data of cVNS and taVNS

**Fig. 3:**
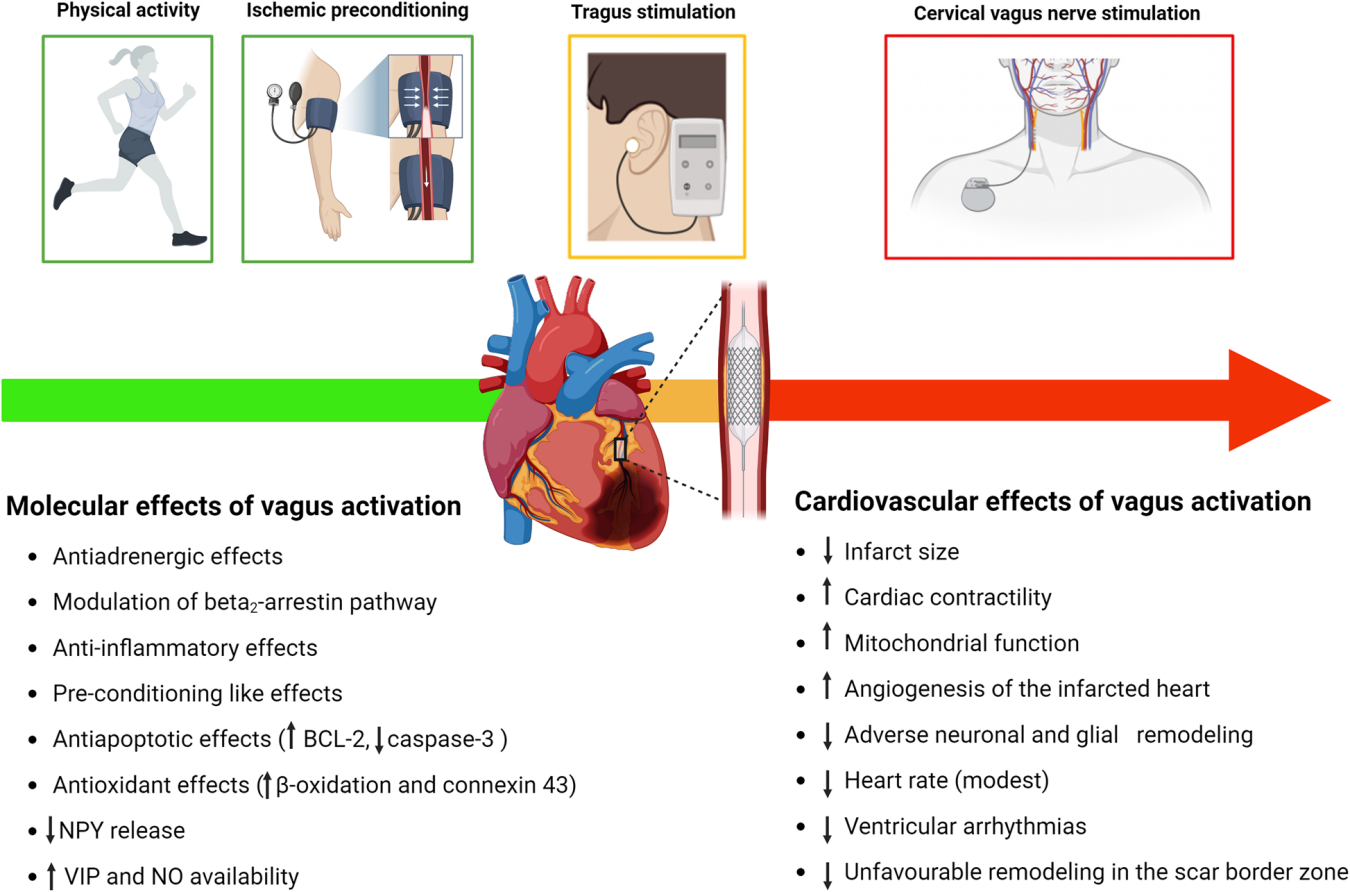
Potential approaches to increase cardiac vagal output before (green), during (yellow) and after (red) myocardial ischemia and reperfusion. NPY Neuropeptide Y; VIP: Vasoactive intestinal peptide. Modified from Dusi et al. 2023

Overall, pre-clinical data consistently showed a strong cardioprotective effect against acute and chronic cardiac injuries of both cVNS and taVNS (Elamin et al. 2023), even when applied at a low-level, meaning at a stimulation intensity unable to evoke acute HR changes. In the setting of myocardial I/R damage, the earlier the application, the greater the benefit (Dusi et al. 2023). No study has ever directly compared in the same model the amount of benefit of the two techniques, that do share the same final pathway represented by an improved autonomic balance (Dusi and Ardell 2020). The main unraveled mechanisms underlying the previously described beneficial effects of cVNS at the cardiac level (Fig. 3) include a direct antiadrenergic effect (both at the central and at the peripheral level, pre-synaptic and post-synaptic) that encompasses a HR-lowering effect, pre-conditioning like effects, anti-apoptotic and antioxidant effects, pro-angiogenic and anti-inflammatory effects (Dusi and Ardell 2020; Dusi et al. 2020). The majority of these effects, as well as the underlying molecular pathways, have already been confirmed as the bases for the cardioprotective actions of taVNS (Table 2); the remaining, that include the metabolic and the mitochondrial effects, still lack a direct demonstration at the cardiac level but were consistently associated with the cerebral protection provided by taVNS against neuronal I/R damage. Since both cVNS (Frangos et al. 2015; Shen et al. 2011; Komisaruk and Frangos 2022) and taVNS (Wang et al. 2014) were proved to reduce cardiac sympathetic output, a reduced released of the proarrhythmic and vasoconstrictor NPY, that, as already mentioned, is co-released by sympathetic neurons in the setting of high-level activation, could be inferred. The first, direct confirmation that cVNS can reduce in vivo NPY levels in the ventricles during bilateral stellate ganglia stimulation in healthy pigs was only recently reported (Jani et al. 2024) and associated with the activation of presynaptic sympathetic muscarinic receptors.

**Table 2:** Summary of the proven beneficial effects of cVNS and of taVNS (including the molecular bases) on the different pathophysiological pathways involved in the I/R damage

**Pathophysiological pathway involved in the I/R damage**	**Proven beneficial effects of cVNS at the cardiac level**	**Proven beneficial effects of taVNS at the cardiac level**
Adverse metabolic changes	Shift in cardiac fatty acid metabolism toward mitochondrial beta oxidation (Nuntaphum et al. 2018). Functional preservation of Cx43 (Sabbah et al. 2011; Shinlapawittayatorn et al. 2014; Nuntaphum et al. 2018; Ando et al. 2005)	Still unproven (but demonstrated for neuronal I/R damage via peroxisome proliferator-activated receptor-*γ* (PPAR-*γ*) activation (Li 2020))
Mitochondrial dysfunction	Attenuated cardiac mitochondrial dysfunction (ROS production and mPTP opening) (Shinlapawittayatorn et al. 2014) and improved mitochondrial dynamic (Nuntaphum et al. 2018).	Still unproven (but demonstrated for neuronal I/R damage (Kim et al. 2022))
Cell death	Specific cardioprotective effects due to the upregulation of the anti-apoptotic protein BCL-2 and the suppression of caspase-3 via phosphatidylinositol 3-kinase and Akt mediated pathways (Kakinuma et al. 2005; Uemura et al. 2010; Mioni et al. 2005).	General cardioprotective effects (Wang et al. 2014) (associated with (PPAR-*γ*) activation (Li 2020) in the setting of neuronal I/R damage) (Melo et al. 2023)
Inflammation and microvascular obstruction	Activation of the cholinergic anti-inflammatory pathway (CAP) at the cardiac (Calvillo et al. 2011) as well as extracardiac level (Tracey 2002; Sabbah et al. 2011; Zhang et al. 2009) Increased NO availability (Allen et al. 2022) Direct vasodilatory effects of neuronally released VIP (Feliciano and Henning 1998; Henning and Sawmiller 2001; Feliciano and Henning 1998; Shanks et al. 2023) Reduced release of NPY (potent vasoconstrictor thought Y1 (Herring et al. 2019) and Y5 type (Malmström 2002) receptors) by post-ganglionic sympathetic fibers (Jani et al. 2024)	Systemic (Wang et al. 2014) and cardiac (Zhou et al. 2019) anti-inflammatory effects Pro- angiogenetic effects of taVNS proven against neuronal I/R injury (Baig et al. 2022; Li 2020)
Abnormal cardiovascular afferent signaling	Increase in the afferent signaling traveling through protective vagal pathways (direct effect) (Weperen and Vaseghi 2023; Vaseghi, et al. 2017; Schwartz et al. 1973) and decrease in the one traveling through the sympathetic ones (indirect effect) (Vaseghi, et al. 2017).	Increase in afferent vagal signaling (direct effect) (Frangos et al. 2015)
Reduced cardiac vagal output	Increase in cardiac vagal output (direct and/or indirect (Schwartz et al. 1973) effect, depending on the stimulation protocol) (Ardell et al. 2017)	Increase in cardiac vagal output (indirect effect) (Frangos et al. 2015; Zhou et al. 2019; Hua et al. 2023)
Increased cardiac sympathetic output	Antagonism of sympathetic activation at multiple levels: - Central (direct) (Frangos et al. 2015; Shen et al. 2011; Komisaruk and Frangos 2022) - Peripheral (Schwartz et al. 1973; Hoang et al. 2022)(direct, pre- and post-synaptic)	Antagonism of sympathetic activation at multiple levels: - Central (direct) (Frangos et al. 2015) - Peripheral (Indirect) (Wang et al. 2014; Yu et al. 2016)
Unfavorable structural autonomic remodeling	Attenuation of the unfavorable structural remodeling in the left stellate ganglia and in the dorsal root ganglia (Hadaya et al. 2023) (affecting both neurons and glial cells)	Attenuation of the unfavorable structural remodeling (sympathetic neuronal sprouting) within the myocardium and the left stellate ganglia (Yu et al. 2016).

As already underlined, cVNS directly delivers a mixed (afferent and efferent) stimulation, with the relative afferent and efferent contribution depending on the electrode characteristics and the stimulation protocol (Dusi and Ferrari 2021), while taVNS directly conveys a purely afferent stimulation. Additionally, cVNS affects most vagal afferent fibers (including all the cardiac ones), while taVNS only those terminating at the auricular level. Therefore, from a theoretical point of view, cVNS would be expected to be more effective than taVNS. Yet, it must be remembered that, lacking a direct efferent effect, taVNS might be a more physiological way to increase cardiac vagal output because the central integration leading to the improved autonomic balance is preserved (as for BAT). Accordingly, the entire rationale of the “neural fulcrum” (Ardell et al. 2017) concept proposed by Ardell and colleagues for cVNS relies on the concept that the best way to achieve cVNS is the one not acutely leading to a significant HR change while chronically preserving the physiological circadian HR pattern. Table 3 summarizes the main studies assessing the effects of cVNS in myocardial I/R injury and in post-cardiac arrest syndrome and the effects of taVNS in the setting of acute (within 24 hours) myocardial ischemia.

**Table 3:** Main studies assessing the effects of cVNS in myocardial I/R injury and in post-cardiac arrest syndrome and the effects of taVNS in the setting of acute (within 24 hours) myocardial ischemia. cVNS: cervical vagal nerve stimulation, I/R: ischemia/reperfusion injury, LAD: left anterior descending artery, taVNS: Trancutaneous auricular vagal nerve stimulation

Study	Year	Animals used	CV Disease	Study protocol	Device	Outcome/Results
Shinlapawittayatorn, K. *et al.*	2013	32 pigs randomized into 4 groups	I/R injury (1 h of I/120 minutes of R) obtained through LAD ligation.	Left cVNS (20 Hz, 3.5 mA, 500 μs pulse width) was applied at the onset of ischemia and throughout the end of reperfusion, either intermittently (cycles of 21-sec ON and 30-sec OFF) or continuously. One group only had I/R injury (sham operated), another had intermittent-cVNS+atropine. The effects were assessed at the end of reperfusion.	Generator: Demipulse, Model 103, Cyberonics Electrode: Bipolar electrode (Model 304, Cyberonics).	Left cVNS significantly reduced infarct size, improved LV function, decreased VF episodes, and attenuated cardiac mitochondrial ROS production, compared with the control group. The amount of benefit, particularly of infarct size and VF episodes reduction, was greater with intermittent cVNS. These beneficial effects were abolished by muscarinic blockade.
Shinlapawittayatorn, K. *et al.*	2014	28 pigs randomized into 4 groups	I/R injury (1 h of I/120 minutes of R) obtained through LAD ligation	Intermittent left cVNS (20 Hz, 3.5 mA, 500 μs, 21-sec ON, 30-sec OFF) was applied during late ischemia (30 minutes after LAD occlusion) or at the onset of reperfusion. One group only had I/R injury (sham operated), another had cVNS during ischemia plus atropine. The effects were assessed at the end of reperfusion	Generator: Demipulse, Model 103, Cyberonics Electrode: Bipolar electrode (Model 304, Cyberonics)	Left cVNS started during late ischemia, but not at reperfusion, still retained beneficial effects on electrical stability, infarct size, cardiac function, inflammatory biomarkers, ROS production and phosphorylated Cx43 levels. These beneficial effects of cVNS were abolished by atropine.
Nuntaphum, W. *et al.*	2018	30 pigs ramdomized into 5 groups	I/R injury (1 h of I/120 minutes of R) obtained through LAD occlusion	Intermittent left cVNS (20 Hz, 3.5 mA, 500 μs, with continuously recurring cycles of 21-second ON, 30-second OFF) was applied to 4 groups: 1) with both vagal trunks intact (LC-VNS), 2) with left VN transection (LtVNX, selective left efferent stimulation), 3) with right VN transection (RtVNX) and 4) with atropine pretreatment, respectively. A fifth group only had I/R injury with no left cVNS (control group). The effects were assessed at the end of reperfusion	Generator: Demipulse, Model 103, Cyberonics Electrode: Bipolar electrode (Model 304, Cyberonics)	LC-VNS and LtVNX produced a similar and more profound cardioprotective effect (using infarct size as primary endpoint, an 89% reduction for LC-VNS and an 84% for LtVNX was observed) compared to RtVNX (63% reduction).
Wang, Q. *et al.*	2015	100 Sprague Dawley rats ramdomized into 5 groups	I/R injury (30 min of I/120 minutes of R) obtained through LAD ligation Noninvasive limb remote ischemic perconditioning (RIPerC) employs brief limb ischemic stimuli during myocardial ischemia and before reperfusion	There were 3 treatment groups: Continuous right cVNS (10 Hz, 2-ms, amplitude set to reach a 10% acute decrease in HR) alone applied at 15 min of ischemia and lasting for 30 minutes, cVNS (same characteristics as before) associated with RIPerC, and RIPerC alone. One group did not receive I/R, another only had I/R injury (in both cases with VN exposure). Serum enzymatic markers, serum inflammatory cytokines, myocardial inflammatory cytokines, and infarct size were assessed	Generator: BL-420 stimulator, Technology & Market, Co. Electrode: Bipolar platinum electrodes	The cardioprotective effects of right cVNS applied as perconditioning (during ischemia) in combination with limb remote ischemic perconditioning were superior to those achieved with either treatment alone (cVNS alone or RIPerC alone),
Kim, S. *et al.*	2022	54 Sprague-Dawley rats randomized into 2 groups	Acute post-cardiac Arrest Syndrome in an asphyxial cardiac arrest model obtained through vecuronium	Left cVNS (1 Hz, 10 ms, 1 mA) applied for 3 h after ROSC was compared with the sham control group (device application with no active cVNS)	Generator: Model 2100, A-M Systems, Sequim. Electrode: Plexiglas-platinum electrode, 73-0336, Harvard Apparatus, Holliston.	Left cVNS improved mitochondrial dysfunction and neurological outcomes at 48 and 72 h during post-cardiac arrest syndrome
Choudhary, RC. *et al*	2022	16 Sprague-Dawley rats randomized into 2 groups	Acute post-cardiac Arrest Syndrome in an asphyxial cardiac arrest model (vecuronium)	Intermittent (duty cycle 25%, 10 sec ON, 30 sec OFF) left cVNS (30 Hz, 500 μs, amplitude set to reach a 15-20% acute HR drop) applied for 2 h after ROSC was compared with the sham control group (device application with no active cVNS)	Generator: STG4008 stimulator (Multichannel Systems)	Left cVNS improves neurological outcomes at 24, 48 and 72 h and attenuates neuronal damage in the hippocampal CA1 region, in heart and kidney after cardiac arrest and resuscitation
Sun, P. *et al.*	2018	40 m Sprague–Dawley rats randomized into 4 groups	Acute post-cardiac Arrest Syndrome obtained by ventricular fibrillation induction	Left cVNS (10 Hz, 500 μs, amplitude set to reach a 10% acute HR drop) applied for 4 h after ROSC was compared with the sham control group (device application and CPR with no active cVNS), with the group who received left cVNS+ **α**7nAChR antagonist MLA and with the group who only received the **α**7nAChR agonist GTS-21 (without cVNS).	Generator: Model 8002A pulse generator; Hewlett Packard.	HR was reduced in the cVNS and cVNS+MLA groups, while no difference was found in mean arterial pressure between the four groups. Better post-resuscitation myocardial and cerebral function and improved survival were observed in the cVNS-treated animals. The protective effects of VNS could be abolished by MLA and imitated by GTS-21. In addition, VNS decreased the number of electrical shocks and the duration of CPR required.
Wang, Z. *et al.*	2014	30 beagle dogs randomized into 3 groups	Acute myocardial ischemia and subsequent myocardial infarction obtained through LAD ligation	Bilateral intermittent low-level taVNS at the tragus level (20 Hz, 1 ms, at 80% of the acute HR lowering threshold, duty cycle: 50%, 5 sec ON and 5 sec OFF) was delivered 4 hours per day (from 7 to 9 AM and from 4 to 6 PM) after coronary ligation, for 90 days. One group only received coronary ligation, the third sham surgery without stimulation. The effects were assessed after 90 days.	Generator: Custom-made Electrodes: ear clips	After a 90-day period follow-up, chronic taVNS significantly reduced LA and LV dimensions, improved LV systolic and diastolic function and reduced infarct size by ≈50% compared with MI group. These beneficial effects were accompanied by reduced levels of profibrotic factors, hs-CRP, norepinephrine and NT-proBNP starting from the 7th day to the end of follow-up.
Yu, L. *et al.*	2016	22 beagles dogs randomized into 3 groups	Acute myocardial ischemia and subsequent myocardial infarction obtained through LAD ligation below its first diagonal branch	Right intermittent low-level taVNS at the tragus level (20 Hz, 1 ms, at 80% of the acute HR lowering threshold, duty cycle: 50%, 5 sec ON and 5 sec OFF) was delivered 2 hours per day after coronary ligation, for 2 months. One group only received coronary ligation, the third sham surgery (LAD separation without ligation) without stimulation. The effects were assessed after 90 days.	Generator: Custom-made Electrodes: ear clips	Chronic right-sided taVNS reduced VAs inducibility, decreased cardiac sympathetic output and attenuated cardiac sympathetic remodeling induced by chronic MI compared to controls. Infarct size was not assessed in this model.

## Clinical data on taVNS in myocardial I/R injury

As already mentioned, taVNS may overcome the issues related to the use of an invasive method, particularly in the acute setting, while retaining the advantages of invasive cVNS.

In humans, taVNS has already been tested in several non-cardiological conditions including neurological disorders (epilepsy, depression, migraine, tinnitus, anxiety, mild cognitive impairment, chronic stroke) (Baig et al. 2022; Zhu et al. 2022; Tan et al. 2023) and inflammatory disorders (acute COVID-19 infection (Uehara et al. 2022) and long COVID (Badran, et al. 2022)). Specifically, pilot clinical trials of taVNS show that taVNS paired with rehabilitation may improve upper limb motor and sensory function in patients with chronic stroke (Baig et al. 2022). At present, there are several registered randomized clinical trials of taVNS in stroke (Baig et al. 2023).

In the cardiovascular area, most clinical studies with taVNS were performed in the setting of atrial fibrillation and of HF with both reduced and preserved LVEF, with promising results (Elamin et al. 2023); notably, in all studies a consistent reduction in inflammatory markers was observed (Elamin et al. 2023). As already mentioned, the Parasym device has been the most studied in these settings so far (Elamin et al. 2023). Concerning the stimulation protocols, all cardiac studies used a low-level (unable to acutely affect HR) mode of taVNS delivery, mostly at a 20 Hz pulse frequency and a 0.2 msec pulse duration, and with a current amplitude mostly titrated up to a 1 mAmp below the discomfort threshold (typically in the range of 10–50 mA). Yet, the overall daily duration of the taVNS was extremely variable, from a minimum of 1 hour in outpatients with atrial fibrillation (Stavrakis et al. 2020) to a maximum of 8 hours (4 hours twice daily, between 6 AM-10 AM and 6 PM −10 PM) in hospitalized patients with acute decompensated HF (Dasari, et al. 2023).

Specific clinical experiences in the setting of acute and chronic coronary artery disease are very limited. Back in 2001, Zamotrinsky et al (2001). were the first to report, in a small, randomized study including a total of 18 patients, about a custom-made form of taVNS for the treatment of patients with severe refractory angina candidates to coronary artery bypass grafting (CABG). Patients were randomized 1:1 to taVNS or control (with no sham treatment). All patients were male, aged 48–58 years, with stable angina pectoris class IV of the Canadian and Cardiovascular Society classification, with daily anginal attacks at rest and under a low workload as assessed by bicycle exercise testing. None of them had diabetes mellitus or atrial fibrillation; LVEF range was 49–59%. After admission, the patients spent 25–30 days in the hospital awaiting surgery, during which they received a course of 10 stimulation procedures lasting 15 min, repeated for 10 consecutive days. TaVNS was delivered with an impulse current generator connected to a pair of electrodes and attached to short acupuncture needles put into the skin. TaVNS treatment abolished angina at rest, decreased HR and blood pressure, improved LVEF and LV diastolic filling and decreased the QRS as well as the QT interval (with an associated T wave morphology improvement represented by augmented T-wave amplitude in precordial leads). In the postoperative period, the percentage of patients developing HF dramatically decreased from 90% in the taVNS group to 12% in the control group. of patients treated with taVNS. Intriguingly, taVNS was reported to have acute anti-anginal effects like that of glycerol trinitrate. Of note, almost none of the patients was on beta-blockers as per procedure local management in patients with depressed LVEF, and that might have amplified the effects of taVNS. Finally, surgical specimens obtained at the time of surgery showed a higher density of cardiac microcirculatory vessels in the taVNS group compared to controls.

More than 15 years later, in 2017, Yu et al (2017) reported the first and, so far, only, proof of concept clinical study, of taVNS in I/R injury. Patients presenting with sopraST elevation myocardial infarction (STEMI) within 12 hours of symptom and undergoing primary percutaneous coronary intervention, were randomized 1:1 to active right taVNS (*n*=47) or sham-stimulation (*n*=49). Patients with left main or multiple coronary artery disease were excluded. Low-level taVNS was delivered at 50% below the sinus rate lowering amplitude threshold, at a pulse frequency of 20 Hz and pulse duration of 1 ms with a duty cycle of 5 sec on and 5 sec off. Active stimulation or sham control was started as soon as the patients arrived in the cardiac catheterization lab and maintained for 2 hours after reperfusion (balloon dilation); patients were subsequently monitored for the next 7 days. Most patients (75%) were male, 32% had diabetes (not better characterized), 66% had the LAD as culprit artery and the mean ischemia time (from symptoms onset to reperfusion start) was 6 hours. Reperfusion-related ventricular arrhythmias, assessed with 24 hours Holter ECG, were markedly reduced by taVNS, both the isolated PVCs, and the repetitive forms; indeed, ventricular tachycardia episodes were 18±12 in the sham compared to 2±3 in the active group (*p* < 0.05). Inflammatory marker levels at 24 hours after reperfusion (including IL-6, IL-1β, high mobility group-box 1 protein, TNF–α) were also significantly improved, despite no differences at the first blood sampling determination before reperfusion. The area under the curve over 72 h for the markers of myocardial damage (creatine kinase-MB and myoglobin) was smaller in the taVNS group, and that was associated with lower NT-proBNP levels, and better cardiac systolic and diastolic function as assessed by blinded 2-dimensional echocardiography assessment on days 5 to 7 after revascularization (LVEF 52± 64% versus 49± 8%, *p*= 0.01). The main limitation of this study is that it lacks a detailed estimate (by cardiac MRI) of the final infarct size compared to the initial area at risk to further reinforce these findings and provide a better comparison with animal data; also, the troponin levels were not provided. As expected, the feasibility, tolerability and safety profile of the device were excellent since no side-effects nor discontinuation cases were reported. All patients were conscious and were not given any sedatives during taVNS. Notably, as opposed to pre-clinical studies, the usage of taVNS on unconscious patients has never been reported so far. Local electrical capture, central integration of the inputs and peripheral effects are expected to be influenced by the level of consciousness and/or the usage of neurodepressant drugs, and that might be an issue for the taVNS implementation, for instance in the emergency setting of PCAS. On the other side, the feasibility of taVNS in an acute setting with preserved consciousness, such as acute decompensated heart failure (ADHF) was recently confirmed (Dasari, et al. 2023).

## Future prospectives

The non-invasive nature of taVNS, in addition to its strong pathophysiological rationale shared with cVNS, makes it a very appealing treatment option for several conditions including I/R injury. Yet, there are still important issues slowing the clinical transition of both taVNS and cVNS, being the extensive assessment of the dose response relationship the most important one.

A better understanding of the functional anatomical organization of the vagus nerve across species is highly needed and currently pursued through ongoing clinical (cadaveric) and pre-clinical studies using advanced imaging techniques such as micro-computed tomography (Thompson et al. 2020). Despite that, several studies with hundreds of subjects would be required to identify the most suitable electrode configuration and stimulation protocol, with obvious ethical and practical concerns. Computational model strategies (Capogrosso and Lempka 2020) integrated with artificial intelligence (AI) techniques (Gallone et al. 2022) are expected to help overcoming these huge limitations of in vivo studies, and to provide an important drive in the clinical implementation of electrical neuromodulation in the near future. For instance, a multilayer computational model for neurocardiac modulation that includes sympathetic and parasympathetic branches, synaptic dynamics and conductance based integrate-and-fire neurons was recently developed. The pivotal characteristic of the model is that it recapitulates the connection between the autonomic nervous system and both pacemaker and contractile cardiomyocytes (Yang et al. 2023). The model was specifically developed to evaluate the impact of autonomic changes on arrhythmias susceptibility, but a similar model aimed to assess the effects on infarct size, albeit much more complex to realize, is now foreseeable (Liu and Ajijola 2023). Additionally, extending the now well-established health care applications of AI from the clinical (D’Ascenzo et al. 2021; Filippo et al. 2023; Saglietto et al. 2024) to the pre-clinical field (Diaconu et al. 2022) will lead to a tremendous improvement of the mechanistic understanding of the interactions between electric fields and neural circuits, that is still currently incomplete. Applied to taVNS, that includes both the central areas activated by taVNS, as well as the short and long-term impact of taVNS on the VN and on the ICNS structure and function (Giannino et al. 2024). Underpinning the complex connectivity within the ICNS, as well as its real-time changes, is particularly complex, and requires advanced techniques of in vivo neuronal recording and processing and dedicated expertise and laboratories (Mehra et al. 2022).

From a clinical standpoint, the rigorous assessment of the efficacy of taVNS in a multicentric randomized controlled study would require the evaluation of the final infarct size as percentage of the area at risk through cardiac MRI, that is still not easily available in the clinical practice in the acute and post-acute setting (Arai 2011). Universal standardization of cardiac MRI protocols/endpoints is also of the utmost importance to promote the incorporation of cardioprotective interventions into clinical practice (Ibanez et al. 2019). The implementation of novel predictive markers of autonomic dysfunction is also an area of extensive research (Mehra et al. 2022).

Finally, the development of closed loop systems, with the possibility to continuously monitor and integrate a biofeedback and adjust the stimulation parameters accordingly, albeit particularly complex to incorporate in a non-invasive system, is already under evaluation even for taVNS (Yu et al. 2022). Indeed, electrocardiography (ECG)-gated closed loop (CL)-taVNS, at present not commercially available but technically feasible using wearable ECG devices and Bluetooth based networks (Beniwal et al. 2021), might be able to set the intensity of stimulation based on hear rate, heart rate variability and the presence and the severity of arrhythmias. Even mor complex, a subcutaneous humoral signal (SHS)-gated CL-taVNS might also be developed, using as biofeedback circulating biomarkers and cytokines levels (Yu et al. 2022), assessed at a subcutaneous level.

To underscore the importance of sharing experiences, knowledge and technological tools to improve our overall understanding of autonomic nervous system functioning and pave the way for successful neuromodulation strategies, an international consortium named SPARC (Stimulating Peripheral Activity to Relieve Conditions) founded by the NIH, was instituted (Stimulating Peripheral Activity to Relieve Conditions (SPARC) n.d.). All the experimental data and mathematical models continuously supplied by the consortium members are curated, annotated and semantically linked in a single, freely accessible, data portal. The SPARC program has recently begun Phase 2, that includes a new initiative (called SPARC-V) dedicated to the vagus nerve, encompassing the REVA project, aimed at producing high-resolution maps of the human vagus nerve, and the VESPA project, aimed at assessing the effects of a common set of VNS parameters on many different organs including the heart in coordinated clinical studies.

## Conclusions

The pathophysiological rational for therapeutic interventions aimed at improving vagal output in the setting of I/R injury is very strong, due to the anti-adrenergic, anti-inflammatory, antioxidants, anti-apoptotic and pro-angiogenic effect that finally leads to cardioprotection, reduced infarct size and reduced arrhythmic risk. Transcutaneous auricular VNS may overcome the limitations related to invasive cervical VNS, particularly in the acute setting, but more data are needed to better characterize the dose-effect relationship and the most suitable candidates. A better understanding of the functional and anatomical organization of the vagus nerve, combined with computational model strategies integrated with artificial intelligence tools, will provide an important drive in the clinical implementation of electrical neuromodulation in the next future.

## Data Availability

Not applicable.

## Abbreviations

Ach: Acetylcholine
AChRs: ACh receptors
AI: Artificial Intelligence
ANS: Autonomic nervous system
BAT: Baroreflex activation therapy
CAP: Cholinergic anti-inflammatory pathway
CL-taVNS: Closed loop transcutaneous auricular vagus nerve stimulation
CNS: Central nervous system
CPR: cardiopulmonary resuscitation
cVNS: Cervical VNS
DAMPs: Damage-associated molecular pattern molecules
HF: Heart failure
ICNS: Intrinsic Cardiac Nervous System
IL-: Interleukin
I/R: Ichemia/Reperfusion
LAD: Left anterior descending artery
LSG: left stellate ganglion
mPTP: Mitochondrial permeability transition pores
NCX: Na/Calcium exchanger
NPY: Neuropeptide Y
PCAS: Post cardiac arrest syndrome
PPAR-*γ*: Peroxisome proliferator-activated receptor-*γ*
ROS: Reactive oxygen species
ROSC: Return of spontaneous circulation
STEMI: ST elevation myocardial infarction
taVNS: Transcutaneous auricular vagus nerve stimulation
TLRs: Toll-like receptors
TNFα: Tumor necrosis factor alpha
TNFRs: Tumor necrosis factor receptors
VIP: Vasoactive intestinal peptide
VN: Vagus nerve
VNS: Vagus Nerve stimulation
α7nAChR α7: Nicotinic Cholinergic Receptor
